# Dengue Fever Surveillance in India Using Text Mining in Public Media

**DOI:** 10.4269/ajtmh.17-0253

**Published:** 2017-10-23

**Authors:** Andrea Villanes, Emily Griffiths, Michael Rappa, Christopher G. Healey

**Affiliations:** 1 Department of Computer Science, North Carolina State University, Raleigh, North Carolina;; 2 Public Health England, Sheffield, United Kingdom

## Abstract

Despite the improvement in health conditions across the world, communicable diseases remain among the leading mortality causes in many countries. Combating communicable diseases depends on surveillance, preventive measures, outbreak investigation, and the establishment of control mechanisms. Delays in obtaining country-level data of confirmed communicable disease cases, such as dengue fever, are prompting new efforts for short- to medium-term data. News articles highlight dengue infections, and they can reveal how public health messages, expert findings, and uncertainties are communicated to the public. In this article, we analyze dengue news articles in Asian countries, with a focus in India, for each month in 2014. We investigate how the reports cluster together, and uncover how dengue cases, public health messages, and research findings are communicated in the press. Our main contributions are to 1) uncover underlying topics from news articles that discuss dengue in Asian countries in 2014; 2) construct topic evolution graphs through the year; and 3) analyze the life cycle of dengue news articles in India, then relate them to rainfall, monthly reported dengue cases, and the Breteau Index. We show that the five main topics discussed in the newspapers in Asia in 2014 correspond to 1) prevention; 2) reported dengue cases; 3) politics; 4) prevention relative to other diseases; and 5) emergency plans. We identify that rainfall has 0.92 correlation with the reported dengue cases extracted from news articles. Based on our findings, we conclude that the proposed method facilitates the effective discovery of evolutionary dengue themes and patterns.

## INTRODUCTION

Communicable diseases remain among the leading mortality causes in many countries, particularly in Asia and Africa.^1^ In 2010, of the 52.8 million deaths in the world, 24.9% were due to communicable, maternal, neonatal, and nutritional causes. Moreover, 76% of premature mortality in sub-Saharan Africa in 2010 were due to the same causes.^1^

Combating communicable diseases depends on surveillance, preventive measures, outbreak investigation, and the establishment of control mechanisms.^2^ *Public health surveillance* is the process of monitoring trends through data collection, collation, analysis, and dissemination of public health information for evaluation and public health response, to reduce morbidity and mortality.^3–5^ *Prevention* is the long-term approach to reduce risk factors of a disease burden,^6^ *outbreak investigation* establishes the existence of an outbreak and identifies the source, and *control mechanisms* are meant to cease the spread of a disease to stop its transmission.^7^

Unfortunately, data from surveillance systems are often delayed and reporting is inaccurate, making it difficult to use such data for the detection of outbreaks.^8–12^ Moreover, it is estimated that only 35% of communicable disease cases are reported to national health departments.^13–15^ Underreporting of these diseases negatively impacts the public health policy makers’ abilities to decrease morbidity and mortality.^13,15,16^ A recent study by Shepard et al.^17^ reported an underestimate of 282 times the number of official reported dengue cases in India for one district under study. Furthermore, national health agencies across the globe publish reports that vary in their timeliness: some agencies report data from the previous week, and some have delays that can range as long as multiple years.^18^ In developing countries, existing networks of surveillance systems are not comprehensive for all regions, and relevant communication between countries is often lacking.^9^ Moreover, only official reported information is used in disease control programs, determining patterns of disease, and conducting epidemiologic investigations. Given these issues, the underreporting of communicable diseases has a direct impact on the public's health.^13,15^

The main objective of this study is to investigate the creation of a surveillance tool for dengue fever by applying text mining cluster analysis on news articles that discuss dengue. News articles normally contain local and recent information and could be used to overcome delays in official health reports, if actionable information can be extracted from their text. More specifically, text mining cluster analysis enables analysts to 1) differentiate topics being discussed in news sources; and 2) uncover the evolution of dengue topics to aid in monitoring dengue trends, assisting experts to reduce its morbidity and mortality. For this work, we collaborated closely with dengue experts that provided domain knowledge and guidance on data collection, methods, results of our analysis, and interpretation of our results.

In summary, we identified five main topics from Asian newspapers discussing dengue: 1) prevention; 2) reported dengue cases; 3) politics; 4) prevention regarding other diseases; and 5) emergency plans. In addition, we created topic evolution graphs for the topics extracted from news articles in Asia and in India. These evolution graphs help us to identify topic peaks. Finally, when we incorporated and compared the main dengue indicators in our analysis, we found that the “reported dengue cases” topic extracted from news articles matched peaks in dengue cases and associated dengue measures, suggesting that topic clustering may be a good prediction of dengue onset.

Our analysis shows that text mining cluster analysis in news articles can successfully detect dengue trends occurring in a specific geographic region. Because of the lack of current data for dengue cases, and a need for surveillance systems, our methodology may be applicable for detecting trends and taking preventative actions. We also discuss the issue attention cycle defined by Downs in 1972,^19^ which can be a limitation to our analysis on news articles. We recommend using text mining cluster analysis as a tool for monitoring trends discussing dengue cases, and detecting peaks in reported cases that can affect entire communities.

## MATERIALS

In this section, we present information about dengue fever, and relevant public health surveillance systems.

### Dengue fever.

Dengue is a mosquito-borne viral disease transmitted to humans through infected *Aedes* mosquitoes, a tropical and subtropical species that can be found throughout the world. The principal symptom of dengue is high-grade fever, and can present with any of the following symptoms: facial flushing, skin erythema, body ache, myalgia, arthralgia, and severe headache.^20^ Dengue spread rapidly during the twentieth century to infect more than 300 million people in 2010.^21^ One in three people live among mosquitoes that transmit the dengue virus, yet there remain major uncertainties over the burden of dengue.^22–26^ New, improved methods for assessing this burden are in critical demand.^27^

### Public health surveillance systems.

Urbanization, population movement, increased global travel, commerce in food and medicinal biologic products, and social and environmental changes are some of the main drivers of the need for global surveillance of communicable diseases. Moreover, in developing countries, it is important to detect communicable disease outbreaks early to reduce the number of deaths, the spread, and the resulting harm. Strong surveillance tools are a necessity for both industrialized and developing countries.^28^

According to Thacker,^11^ “public health surveillance is the systematic, ongoing collection, management, analysis, and interpretation of data followed by the dissemination of these data to public health programs to stimulate public health action.” Data collected from public health surveillance can be used to detect epidemics; identify health problems in a region; estimate the magnitude of a health problem, including geographic information about the events; uncover changes in health practices; monitor an agent’s changes; evaluate control measures; and stimulate research. Public health surveillance is the cornerstone for decision-making, allowing decisions to be made more effectively and in a timely manner.

Several efforts have been conducted by the research community to reduce the gaps in information between surveillance systems sponsored by health ministries, public health institutions, nongovernmental organizations, and multinational agencies. Some of the surveillance systems that mine media sources to detect infectious disease outbreaks include HealthMap,^9,29^ BioCaster,^30^ The Global Public Health Intelligence Network (GPHIN),^31^ MedISy,^32^ and EpiSPIDER.^33^

HealthMap (www.healthmap.org) uses Web-based data sources to perform outbreak detection, creates a real-time surveillance system, and updates news on new and ongoing disease outbreaks.^9^ The data collected by HealthMap includes several online electronic media sources, for example, news sources from aggregators such as Google News, reports from the World Health Organization (WHO), and the Program for Monitoring Emerging Diseases (ProMED)-mail, which is a globally moderated mailing list that relies on reports sent by volunteers that include first-hand reports, news stories, and additional data related to previously posted reports. HealthMap uses text mining approaches to automatically classify the sources by location and disease, then visualizes the results on a geographic map.^29^

BioCaster is a nongovernmental surveillance system that uses ontology-based text mining to detect and track disease outbreaks. The system has four main steps: 1) topic classification; 2) named entity recognition; 3) disease/location detection; and 4) event recognition.^30^

GPHIN is a subscription-based Internet system that incorporates news information for global health surveillance. GPHIN relies on two news aggregators: Factiva and Al Bawaba. GPHIN scans, filters, and categorizes information using a taxonomy of keywords and Boolean syntax. The results are then validated by human analysts. In 2005, GPHIN supplied approximately 40% of the WHO’s early outbreak warning notifications.^31^

MedISys is an automated early-warning system for food and food-borne hazards. This system monitors daily news articles from more than 2,200 news sites in 50 languages. The articles are automatically categorized into predefined multilingual categories if they satisfy category definitions based on Boolean and proximity operators.^32^

EpiSPIDER is a Web-based visualization surveillance system for infectious disease threats. EpiSPIDER uses ProMED, the WHO, European Surveillance Network RSS feeds, and news syndication sites such as Reuters as the sources for their reports.^34^ EpiSPIDER extracts the location for each source, then generates country-level maps for all countries.^33^

A system that analyzes news sources for disease severity trends was created in Pakistan. The system characterizes the severity of dengue outbreaks in Pakistan by using news from six local sources to form input for a Support Vector Machine–based classifier that identifies dengue-related articles. These articles are then used to extract the following features: date, location, number of cases, and number of deaths. A severity index is calculated for each location over a period of time based on a polynomial regression model.^35^

Our tool proposes using text mining cluster analysis to: 1) infer topics being discussed in newspaper articles related to communicable diseases 2) understand the evolution of the topics; 3) detect disease outbreaks, and; 4) understand the information being communicated in news articles related to communicable diseases. The main difference with the other tools described herein, is the use of text mining cluster analysis to extract topics from news articles as our main technique of analysis. The tools described above have used text mining, but as a way to extract and classify features like location and disease based on text. Existing tools are interested in finding a predefined set of topics using keyword matching to perform topic assignment. Critically, and unlike our system, these tools cannot adapt to new topics with manually updating the predefined topic list. Our tool dynamically extracts the appropriate set of topics found in a text corpus, without the need for human intervention.

## METHODS

### Text mining.

Text mining is the process of extracting nontrivial and previously unknown information from large text document collections, converting unstructured text data to a structured matrix form.^36^ This is accomplished by converting documents into vectors in some feature space, for example, converting the text in document *D* into a term vector $D_{j}$. Each entry in $D_{j}$ corresponds to a specific term $t_{i}$, and its value defines the frequency of $t_{i}\in D_{j}$. To identify the terms, several representations can be used. The bag-of-words approach is the most common, where each selected word forms a term (or dimension) in the feature space.

Given the size of text documents, feature selection is an important step in text clustering because of high dimensionality and data sparsity. A data collection contains many terms, but only a small number of these normally occur in any individual document. Several sophisticated local and global methods exist for reducing document dimensionality. Local methods remove unimportant or noninformative words, whereas global methods apply a global dimension reduction to transform all documents identically. Popular local methods include: stemming, which reduces words to their stem; stop word removal, which removes noninformative words; and synonym lists, which identify and reduce synonyms to a common word. Global methods include latent semantic analysis, latent Dirichlet allocation (LDA), and nonnegative matrix factorization that characterize documents in terms of *concepts* and sets of terms that represent a more complex idea discussed in a document.

Several techniques are available to identify information in text, such as classification, clustering, and summarization.^37^ Our approach begins with clustering, an unsupervised learning technique in which patterns (observations, data items, or feature vectors) are assigned into homogeneous groups called clusters. A clustering task involves the following components: 1) problem representation, including feature extraction and/or feature selection; 2) calculation of similarity between observations; 3) application of a clustering algorithm; 4) clusters labeling; and 5) evaluation.^38,39^

Text mining cluster analysis, which is the combination of text mining and cluster analysis, groups together documents with similar topics or topics with similar meaning.^40^ The objective of text mining cluster analysis is to classify documents into groups, or clusters, containing documents that are similar to each other. This allows us to identify the main topics being discussed in the document collection.

### Data collection.

To collect dengue fever news articles discussing the Asian region, we searched the LexisNexis Academic database, an online academic database that accesses more than 15,000 news, business, and legal sources.

Multiple search criteria (Table 1) were used to create a search query. The words dengue, DEN-1, DEN-2, DEN-3, DEN-4, and break bone were submitted as keywords to locate relevant news articles. The timeline we queried was January 1, 2014 to December 31, 2014 for the continent of Asia. The year 2014 was chosen over more recent years because of the fact that the most recent dengue indicators are only available for 2014, not for later years, and even then only for limited regions.

**Table 1:** LexisNexis academic search criteria

LexisNexis academic search criteria	Values
Search terms	dengue OR
	DEN-1 OR
	DEN-2 OR
	DEN-3 OR
	DEN-4 OR
	break bone
Date	January 1, 2014 through December 31, 2014
Source	*Blank* (any source)
Content type	Newspapers
Language	English
Geographic location	Asia

Each of the search queries (one query per month) produced a resulting HTML file. We used Beautiful Soup, a Python package for HTML parsing, to extract and process the raw HTML. This allowed us to transform the file into a dataset where each row corresponds to a news article for a specific month. For each news article, we extracted the article’s full text, the title, the publication date, and the length of the article.

### Text parsing.

We next created a structured representation of information stored in the text documents, known as a term-document matrix (TDM). To create the TDM, the following preprocessing steps were applied: 1) stemming—find the stem or root form of a term, aggregating different terms with the same root as equivalent (e.g., run, runs, running and ran would all stem to the root run); 2) stop word removal—words that are common in the text but do not contribute to any useful semantic context are removed using a stop list (e.g., a, an, the, which). The words specified in the stop list are excluded from parsing.

To transform the document collection into a set of terms relevant to all documents, our stop word list was extended to include all the city names in Asia, “dengue”, “dengue fever”, and “break bone”.

The TDM quantitatively represents information contained in a set of documents, by enumerating the frequency of the terms contained in each document.^39^ This converts document *D* into a term-vector $D_{j}$. Each entry $D_{i,j}\in D_{j}$ corresponds to the number of occurrences (the frequency) of a specific term $t_{i}\in D_{j}$. The result of this step is an m×n TDM corresponding to *m* unique terms across *n* documents. Text parsing not only produces a TDM, but also reduces the total number of terms, improving efficiency, and better capturing the content of a document by aggregating terms that are semantically similar.

### Term vector weighting.

We next construct a weighted TDM by applying term frequency–inverse document frequency (TF-IDF). TF-IDF is an algorithm that gives greater weight to terms that occur more frequently within a document (TF), but infrequently across the document collection (IDF). Intuitively, TF-IDF implies that if a term $t_{i}$ occurs frequently in a document $D_{j}$, it is an important term for characterizing $D_{j}$. Moreover, if $t_{i}$ does not occur in many other documents, it is an important term for distinguishing $D_{j}$ from other documents. Given an *n* document collection,

$${\text{TF}-\text{IDF}}_{i,j}=f_{i,j}*\log\left(n-n_{i}\right)$$ (1)

where $n_{i}$ is the number of documents containing term $t_{j}$. This formula varies with the importance of terms based on how frequently the terms occur in individual documents, and how the terms are distributed throughout the document collection. As a final step, each $D_{j}$ is normalized to remove the influence of document length from the TF-IDF weights because longer documents would have higher TF-IDF scores versus short documents.

The weighted TDM becomes the underlying representation for the collection of documents. Once documents have been converted into a weighted TDM, vectors can be compared with estimate the similarity between pairs or sets of documents; determine the optimal number of topic clusters and; perform topic clustering.

### Calculating the pairwise cosine similarity matrix.

A matrix with pairwise document cosine similarities was calculated using the weighted TDM. Here, we use the cosine pairwise similarity measure $s_{u,v}$ to quantify the similarity between a pair of text documents.^38,39^ Mathematically, given two documents *u* and *v*, the cosine similarity is calculated as follows:

$$\cos\theta =\frac{D_{u}-D_{v}}{\left[D_{u}\right]\cdot \left[D_{v}\right]}$$ (2)

Because the document vectors are normalized, this reduces Equation (2) to $\cos\theta =D_{u}-D_{v}$. Dot product similarity represents the cosine of the angle between two document vectors. As the angle between the document vectors nears zero, the more similar the documents are assumed to be. Given the formula, the cosine of the angle between two identical documents is one, whereas the cosine of the angle between two completely dissimilar documents is zero. The corresponding dissimilarity (distance) measure $d_{u,v}$ is given by $1-\cos\theta =1-\cos\left(D_{u}-D_{v}\right)$. Dissimilarity $d_{u,v}$ ranges from zero (identical) to one (completely dissimilar). This converts the cosine similarity matrix to a cosine dissimilarity matrix.

### Determining optimal number of topic clusters.

For each month, multiple *k*-means clustering with increasing *k* was performed on the month’s cosine dissimilarity matrix, to determine the optimal number of clusters *k*.

The elbow method, which is used to calculate an optimal number of clusters, was applied. This visual technique consists of running *k*-means for a range of values of *k*, and for each *k*, calculating the within-cluster sum of squares (WCSS) variation.^41^ The chosen *k* value for each month represents a point on the WCSS line where the reduction in total WCSS slows:

Input—a TF-IDF weighted TDM W

Output—the optimal number of clusters *k*

1. Compute pairwise distance (metric = cosine)
2. For *i* = 1 to 10 do
3. Compute *k*-means
4. Calculate centroids
5. Compute Euclidean distance between the centroids
6. Calculate WCSS
7. Plot WCSS versus *k*
8. End for
9. Locate location of a bend (knee) in the plot.
10. Return *k*

### LDA.

After determining the optimal number of topic clusters, we chose to use LDA to determine the topics in the document collection. LDA is widely adopted to infer topics from text collections, and is best at learning topics from unstructured text.^42^ LDA is an unsupervised topic modeling algorithm, designed to uncover topics (sets of related words) from documents. LDA implements a three-level hierarchical Bayesian model, with each item (document) of a collection represented as a finite mixture over a latent set of topics. LDA assigns a document to a mixture of topics, to characterize the document as a set of associated topic-membership probabilities. This set of topic probabilities is considered an explicit representation of the document.^43^

### Cluster identification labeling.

After obtaining the topics using LDA for each month, a set of describing terms was used to label each topic. This process allows us to describe a topic, and name our final clusters. With the help of a dengue knowledge expert, a label was manually chosen for every cluster for each of the 12 months in 2014 by using the descriptive terms derived by the LDA algorithm. After calculating the optimal number of topics for each month in 2014 using the elbow method described above, a total of eight different topics were identified in the news articles throughout all of 2014.

### Validation.

To validate our LDA clustering technique, we performed training and validation in a set of observations. First, we divided all 3,844 observations into two sets: one for training and one for validation. We trained our model on 3,748 observations and validated the result using the remaining 96 observations. For the validation set, we manually labeled each observation with one of the eight topics that were previously identified. We then applied our LDA model to predict topics for the same 97 validation observations. This allowed us to compare the performance of the model versus manual human topic selection.

As a further comparison of LDA versus other topic clustering approaches, we performed clustering of all 3,844 observations into eight topics using *k*-means, to assess whether LDA provides better classification performance.

Table 2 compares the results from LDA to *k*-means to assess the performance of LDA versus a simpler clustering algorithm. We wanted to ensure that the added complexity of LDA led to significantly better classification results. Based on the 96 observations in the validation set, LDA outperforms *k*-means in correctly classifying manually labeled observations. The misclassification rate is 30.2% (29 incorrectly classified documents) for LDA (Clopper-Pearson 95% confidence interval [CI]: [21.25%, 40.43%]), whereas for *k*-means it is 44.79% (43 incorrectly classified documents) (Clopper–Pearson 95% CI: [34.63%, 55.29%]). Based on this, we chose LDA as our standard classification method. We next discuss the LDA classification results.

**Table 2:** Validation comparison LDA and *k*-means

Labeled topic	Correctly classified LDA	Incorrectly classified LDA	Correctly classified *k*-means	Incorrectly classified *k*-means
0	10	2	7	5
1	9	3	8	4
2	9	3	3	9
3	10	2	6	6
4	8	4	8	4
5	5	7	10	2
6	6	6	6	6
7	10	2	5	7

LDA = latent Dirichlet allocation. Total observations in the validation set = 96.

## RESULTS AND DISCUSSION

### Data collection.

**Figure 1.**
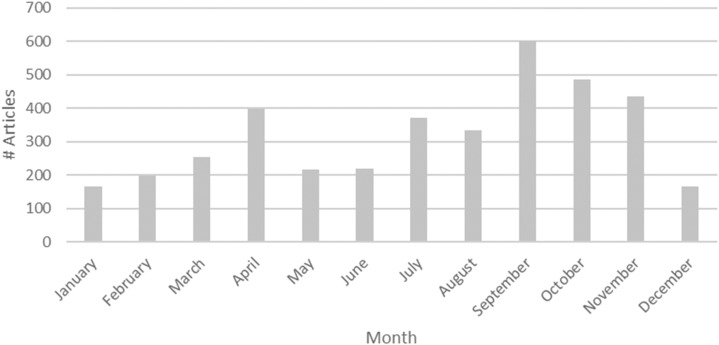
Number of articles collected per month.

After our Lexis Nexis search (Table 1), results were processed to remove duplicates and to infer a country for each observation. A total of 3,844 unique news articles were found. Figure 1 contains the number of dengue articles about Asia collected for each month in 2014.

**Figure 2.**
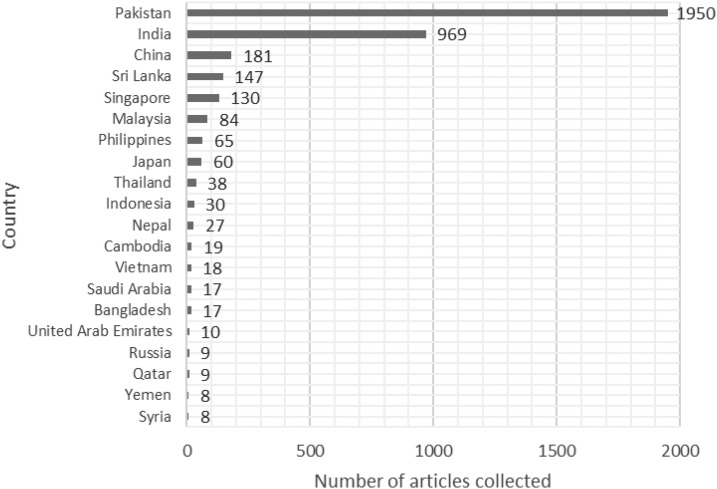
Number of articles collected by country.

**Figure 3.**
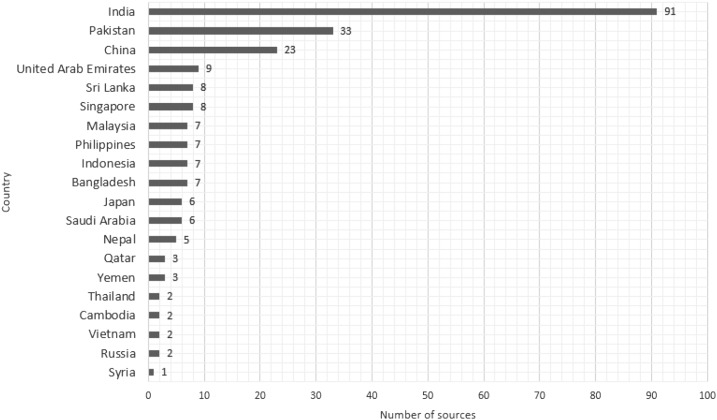
Number of individual sources by country.

The distribution of dengue news articles by country can be found in Figure 2. Non-Asian countries listed in this graph refer to news articles originating in non-Asian countries but where a dengue topic from the continent of Asia was discussed. Figure 3 shows the number of individual sources (indexed by LexisNexis) by country. This can be used to explain why some dengue endemic regions show a low number on the number of articles collected. Furthermore, we can see that India has the highest number of sources indexed by LexisNexis, which allows us to focus in India independently.

**Figure 4.**
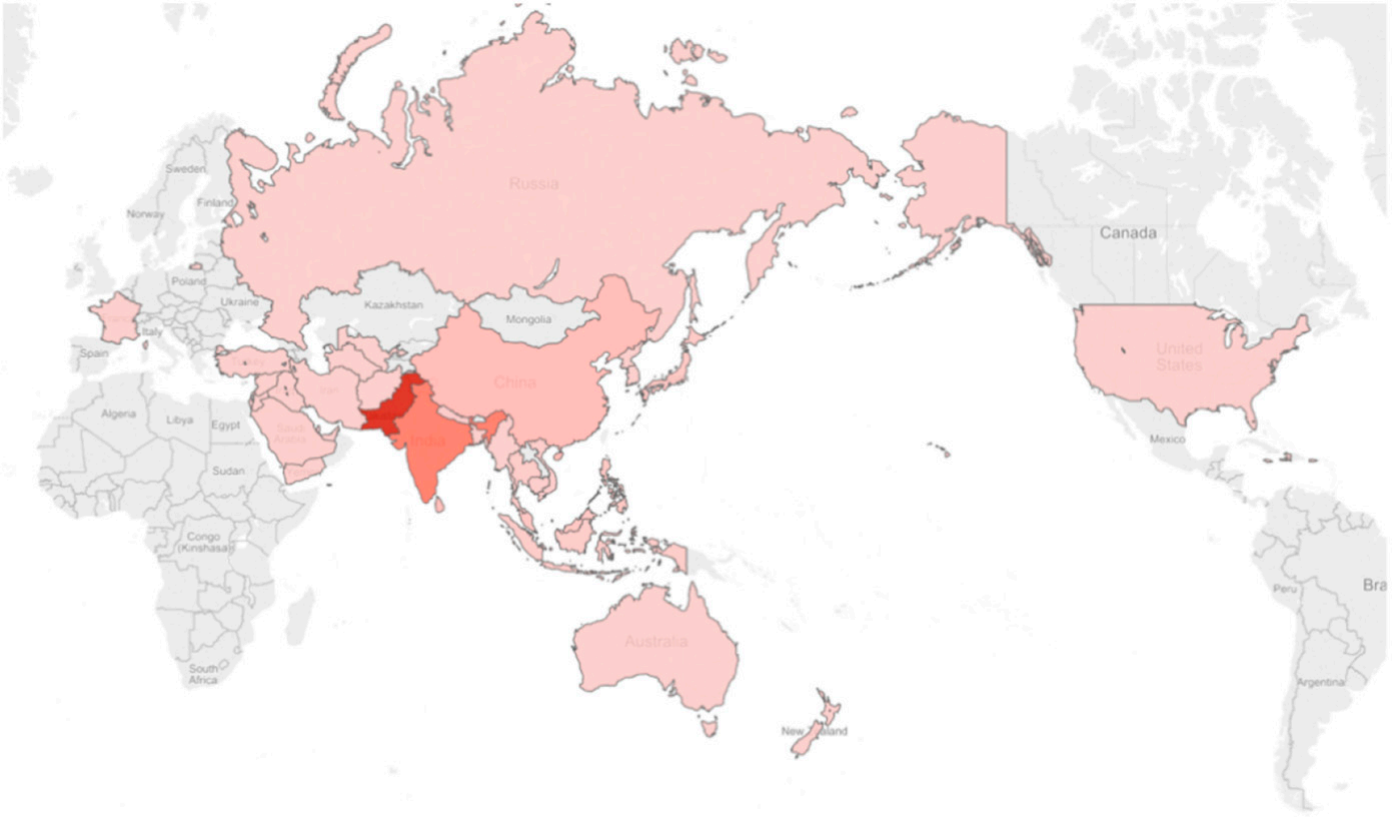
Number of articles collected per country. This figure appears in color at www.ajtmh.org.

Figure 4 visualizes the regions and countries where articles originated. In this figure, the darker the color, the more articles collected from that country.

### Results.

Table 3 details the number of articles discussed for each topic in 2014. The topics were extracted from news articles by running LDA for each of the 12 separate months. The *k* number of topics for each month was chosen using the elbow method. Over all 12 months, the five main topics in articles mentioning dengue in Asia were prevention, reported cases, politics, prevention regarding other diseases, and emergency plan.

**Table 3:** Overall number of articles per topic

Topic label	Number of articles
Prevention	1,546
Reported cases	1,460
Politics	407
Prevention–other diseases	270
Emergency plan	68
Research	42
Vaccines	29
Miscellaneous	22
Total	3,844

**Figure 5.**
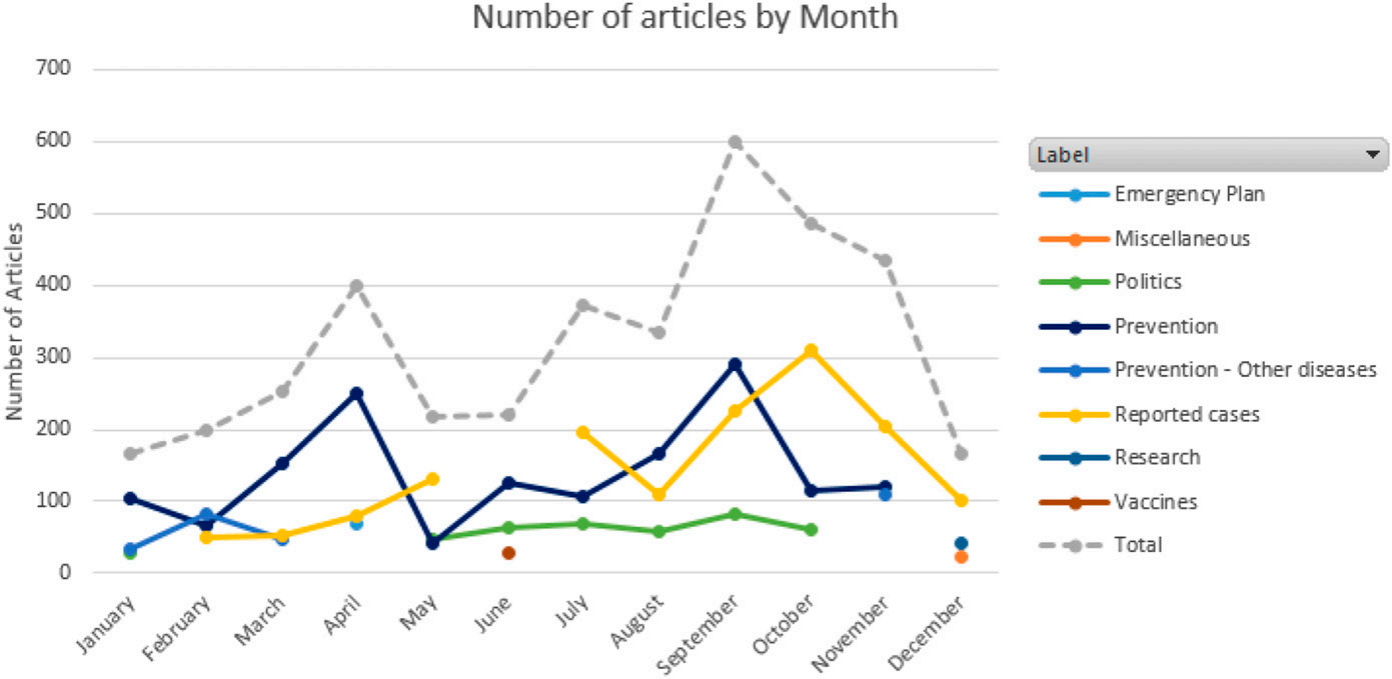
Dengue trends for 2014 in Asia. This figure appears in color at www.ajtmh.org.

Figure 5 visualizes the monthly topic trends found for Asia in 2014. We can see two main peaks—one in April and one in September—in the number of dengue news articles. Based on our topic clustering results, the peak in April is explained because of the increase in news articles discussing prevention (63%), whereas the peak in September is explained because of the increase in news articles that discuss both prevention (49%) and reported dengue cases (39%).

We can also observe that prevention is the only topic discussed throughout most of the year (all months except December), with its primary peaks in April and September. Reported cases have its primary peak in October, with an increasing trend for reported cases from February to May.

Finally, we note that the politics topic starts in May and ends in October, covering the main season of dengue.

### Prediction.

**Figure 6.**
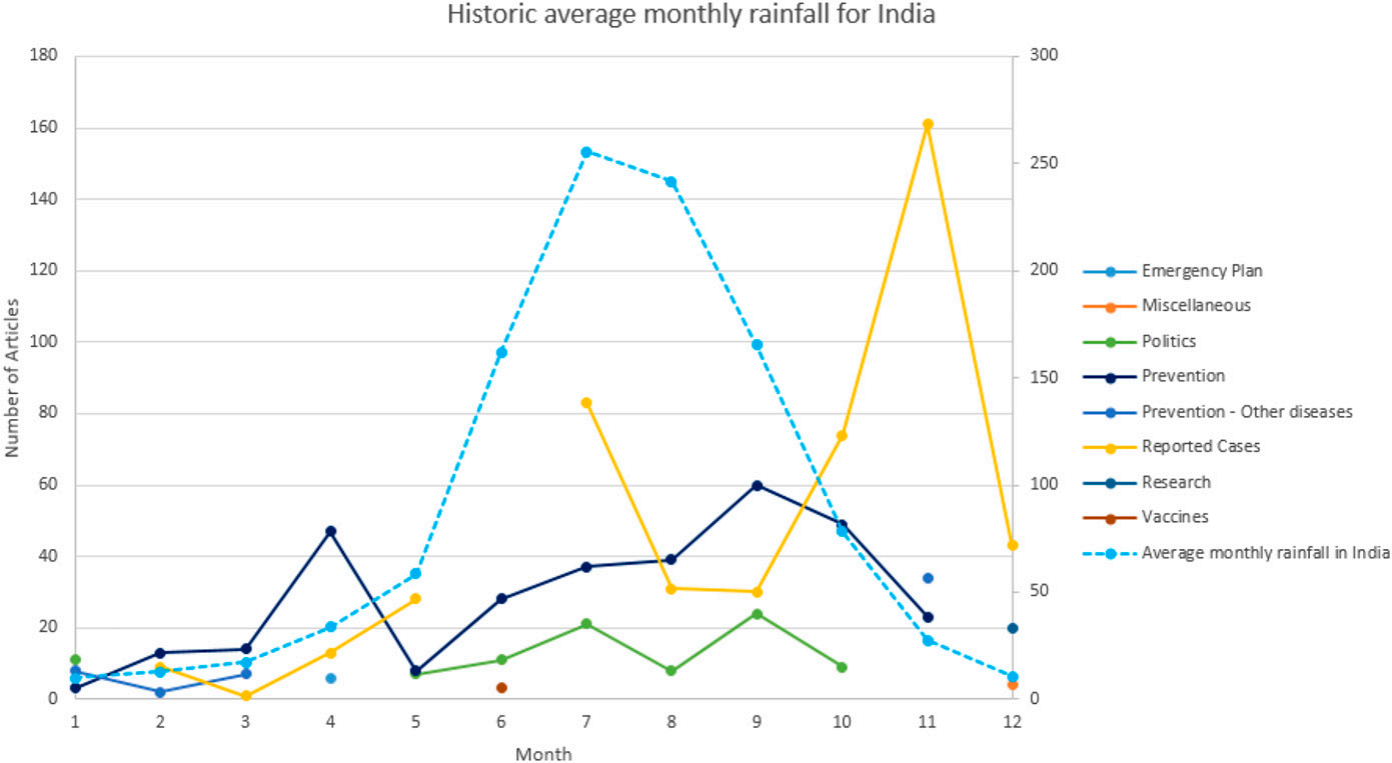
Historic average monthly rainfall for India. This figure appears in color at www.ajtmh.org.

To further explore country-specific details, and to compare our trends with other dengue indicators, we analyzed the country of India separately. Figure 6 shows the topics found in India. Two main trends for 2014 appear: prevention and reported cases. Prevention has two primary peaks in April and in September, whereas reported cases have its primary peaks in July and November. The purpose of comparing our reported cases trend with existing dengue main indicators is to assess how well our reported cases trend can help us predict dengue outcomes.

Historically, researchers have investigated several factors to try to predict dengue cases, such as socioeconomic status and human settlement patterns; migration; temperature and precipitation fluctuations; and Breteau Index levels, which report the number of water containers where dengue is present per 100 houses inspected, documenting the breeding potential of the dengue vector *Aedes aegypti* and *Aedes albopictus*.^44^ Herein, we compare the reported dengue cases extracted from news articles with rainfall and the Breteau Index. We then calculate the correlation between these trends to asses our work.

In Figure 6, we see that the primary peak for average monthly rainfall in India occurs in July, which is the same month where the trend for reported cases starts. The monsoon period for India occurs from June through September, possibly when the dengue vector is more active because conditions favor stagnant water. The June through September period has an average Breteau Index of 8.25, increasing to 21.33 in October, and declining to 0 in February.^45^ Although the Breteau Index can be a strong predictor of dengue outbreaks, it is often not available because it requires a time-consuming and costly individual household inspection.

**Figure 7.**
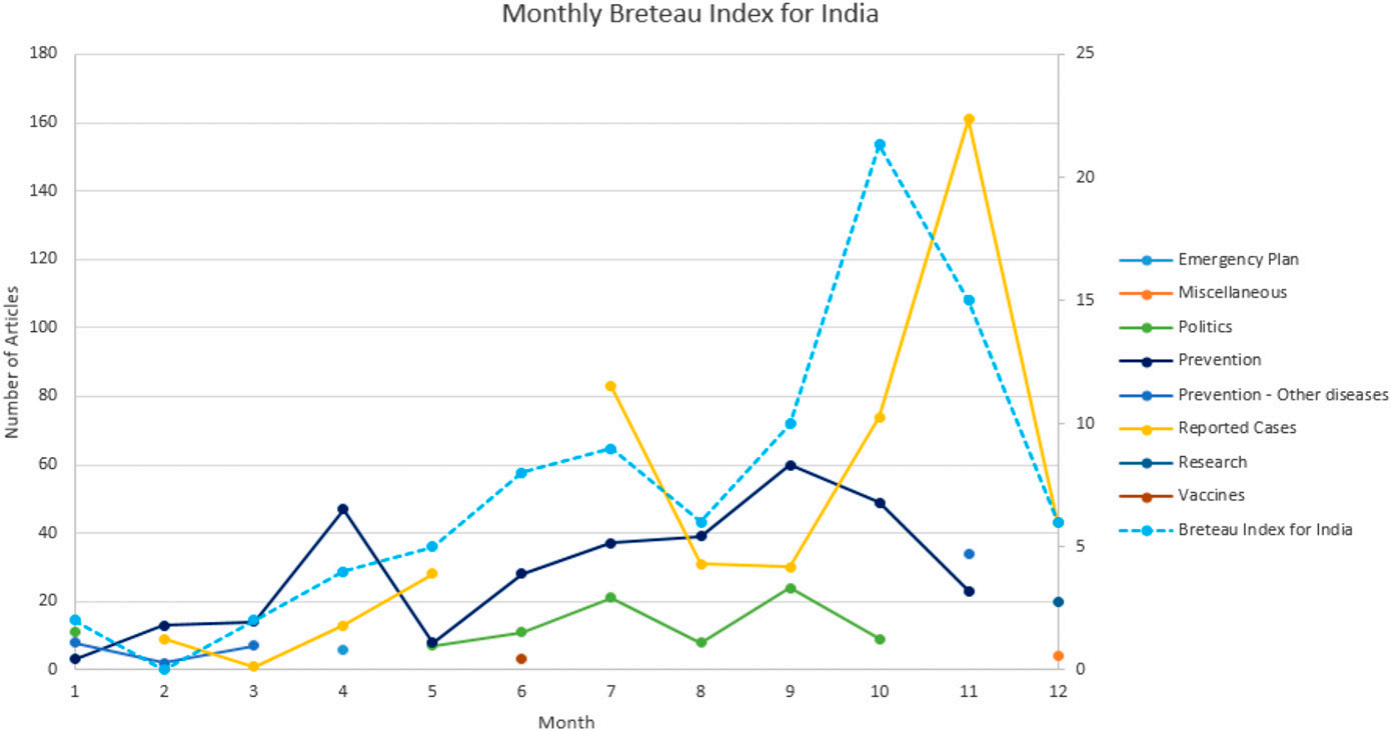
Breteau Index for India. This figure appears in color at www.ajtmh.org.

In Figure 7, the primary peak for the number of news articles on reported cases in India occurs in November, 2 months after the monsoon season declines, and 1 month after the Breteau Index peaks. However, as mentioned before, the Breteau Index is labor intensive and cannot be easily collected in each geographic location.

Finally, the appearance of reported cases aligns with the increase in the precipitation index. Transmission of dengue increases during the monsoon season,^46–49^ as confirmed in our results. Stagnating water after rainfall favors breeding of the mosquito vector, resulting in an increased incidence of dengue.

To the best of our knowledge, official monthly reported positive cases for India in 2014 are not publically available. Because of this, a monthly estimate for the five regions north, south, east, west, and central was calculated for 2014 based on monthly hospital data $\left\{h_{\text{north},\text{Jan}},h_{\text{north},\text{Feb}},\ldots ,h_{\text{north},\text{Dec}}\right\},\ldots ,\left\{h_{\text{central},\text{Jan}},h_{\text{central},\text{Feb}},\ldots ,h_{\text{central},\text{Dec}}\right\}$ found in the literature for each region in India,^50–54^ and the annual 2014 annual number of dengue cases reported by the Government of India $\left\{G_{\text{north}},\;G_{\text{south}},\ldots ,\;G_{\text{central}}\right\}$ for each state. We extrapolated hospital statistics to the state level based on the official annual positive cases reported by the Indian Government as follows for each of the five regions. For example, we used hospital statistics to calculate a January estimate for the north region as

$$\begin{array}{l} H_{\text{north}}=\sum_{i=\text{Jan}}^{\text{Dec}}h_{\text{north},i}\\ h_{\text{north},\text{Jan}\_\text{pct}}=\frac{h_{\text{north},\text{Jan}}}{H_{\text{north}}}\\ G_{\text{north},\text{Jan}}=G_{\text{north}}\times h_{\text{north},\text{Jan}\_\text{pct}} \end{array}$$ (3)

**Figure 8.**
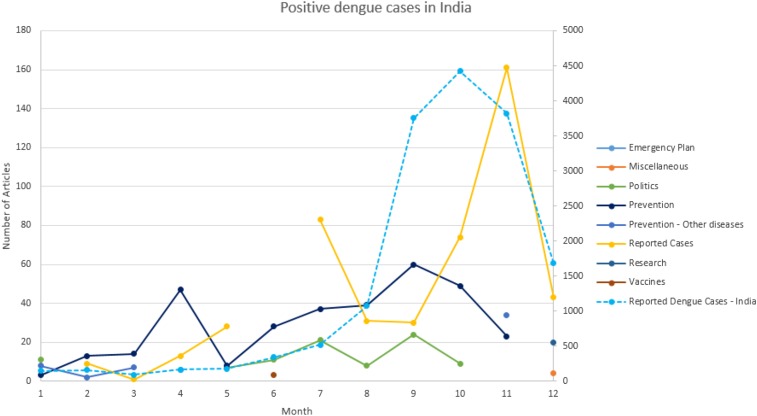
Estimated monthly positive dengue cases in India in 2014. This figure appears in color at www.ajtmh.org.

**Figure 9.**
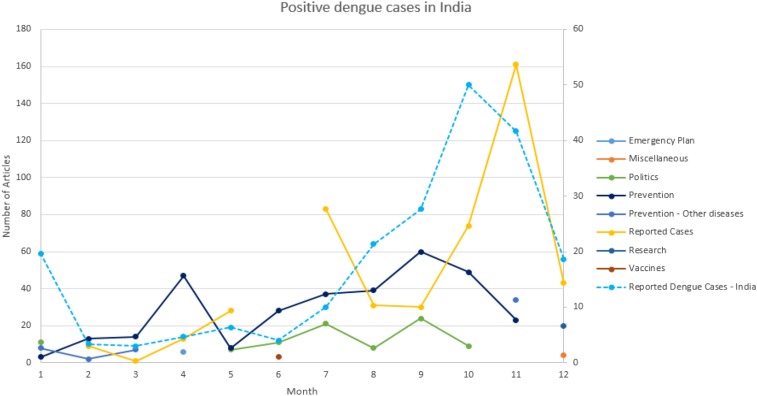
Positive dengue cases in India based on historical data from 2006 through 2008. This figure appears in color at www.ajtmh.org.

Identical calculations were used to estimate cases for the remaining months and four additional regions. Because our methodology does not rely on the number of absolute positive dengue cases, but rather on the trend of positive cases, a monthly estimate for India in 2014 suffices our purpose. In Figure 8, we observe that the estimated number of positive dengue cases in India has its primary peak in October, whereas the news articles reporting dengue cases in 2014 peak in November. We also compared our estimated results with historic data found in the literature from 2006 through 2008 for Chennai.^49^ We observed that the pattern for our estimates and the reported dengue case numbers in Chennai were very similar, as observed in Figure 9. The lack of data of positive reported cases in India for 2014 reinforces our premise that official data are not readily available. Moreover, Singh et al. reported 216 positive dengue cases in one hospital in 2014 for Lucknow in the state of Uttar Pradesh. However, the Government of India reported a total of only 200 positive dengue cases for the entire state of Uttar Pradesh. This report which supports the belief that dengue data are underreported^17^ and new tools to provide surveillance are needed.

The results presented show a correlation between monthly trends of reported cases, rainfall, and the Breteau Index. Correlation is a metric to measure the connection between two variables, ranging from −1 and 1. A correlation of −1 indicates a perfect negative relationship between the two variables (an increase in one produces a corresponding decrease in the other), and a value of 1 indicates a perfect positive relationship. Our results, as indicated in Table 4, show a strong positive correlation (0.92) between the “reported dengue cases” topic extracted from news articles, and the lag of 3 months of average monthly rainfall. In addition, we can see a correlation of 0.69 in relationship with officially reported dengue cases in India with a lag of 1 month. For our prediction purposes, we can infer, given the strong correlation to the “reported dengue cases” topic, that our extracted topics can be used in lieu of dengue trends, such as rainfall and/or official reported cases, supporting the potential usefulness of the proposed methodology in public health surveillance of communicable diseases.

**Table 4:** Spearman correlation coefficients

	Breteau index in India (Lag 1 month)	Average monthly rainfall for India (Lag 3 months)	Official reported dengue cases for India (Lag 1 month)
Reported dengue cases in news articles	0.27	0.92	0.69

### Topic evolution.

In all the dengue trend figures we have presented for Asia and India, we see an abrupt decline in news articles in a given country reporting dengue cases after a peak has occurred. This can be explained by the “issue-attention cycle” identified by Peretz in 1972. He describes how an issue abruptly leaps into prominence (alarmed discovery), remains in strong attention for a short time, and then slowly fades from the center of attention (unresolved most of the time).^19^

This cycle can help us to understand what happens in the trends we see in the dengue news articles: 1) a preproblem stage when dengue prevention takes place, and when the trend for reported cases starts to appear; 2) an alarmed discovery when the number of reported cases peaks; 3) a realization of the cost of significant progress when we start to see the trend of dengue research appearing in the news; 4) a gradual decline in intense public interest after the main peak of reported cases occur; and 5) a postproblem stage in the months of December, January, and February.

## CONCLUSIONS

In this article, we introduce the use of text mining topic clustering to infer topics from news articles discussing dengue; construct topic evolution graphs; analyze the life cycle of dengue news articles in India, and; relate them to rainfall, monthly reported dengue cases, and the Breteau Index. Our work provides the following novel contributions versus existing approaches: 1) topics extracted from news articles offer not only information on dengue trends in a specific geographic area but also information about other topics extracted from news articles, such as prevention, politics, prevention relative to other diseases, and emergency plans; 2) the evolution of topics throughout the year can be used by dengue experts, health care officials, public health policy makers, communicators, and journalists to obtain insight on relationships to a specific communicable disease; 3) although the rainfall and Breteau Index can be used to detect patterns for dengue, this information may not be promptly available, or may not be collected in a specific region. Our proposed methodology can help close the gap and provide reliable information in a specific region; and 4) although interpretation of the clusters may require human input, our analysis can be automated to reduce the delay in receiving official data, and improve the availability of data needed to decrease morbidity and mortality of communicable diseases.

Our future work includes creating a surveillance system for communicable diseases that combines text mining cluster analysis and sentiment analysis. To perform sentiment analysis, we will create a domain-specific sentiment dictionary for communicable diseases. Next, we will study ways to automatically identify sentiment transitions in a given text, to infer the sentiment in the entire document collection. We will investigate ways to visualize the topics and their associated sentiment estimates to identify relationships between topics. Finally, a Web-based tool will be built to facilitate the surveillance of communicable diseases in different regions of the world.
